# Supplementary material for: Successional dynamics of a 35 year old freshwater mitigation wetland in southeastern New Hampshire
Source: PLoS One. 2021 May 17;16(5):e0251748. doi: 10.1371/journal.pone.0251748 (PMC8128273; doi:10.1371/journal.pone.0251748)
Supplement: S1 Table — (PDF) [file pone.0251748.s001.pdf]

| Taxon | 1992 | 2002 | 2020 |
|---|---|---|---|
| PTERIDOPHYTES | | | |
| **DRYOPTERIDACEAE** | | | |
| *Onoclea sensibilis* L. | X | X | X |
| **EQUISETACEAE** | | | |
| *Equisetum arvense* L. | X | X | X |
| **OSMUNDACEAE** | | | |
| *Osmundastrum cinnamomeum* (L.) C. Presl | | X | X |
| *Osmunda regalis* (Willd.) Gray | X | X | X |
| **THELYPTERIDACEAE** | | | |
| *Thelypteris palustris* Schott | X | X | X |
| GYMNOSPERMS | | | |
| **PINACEAE** | | | |
| *Pinus strobus* L. | | | X |
| ANGIOSPERMS - DICOTYLEDONS | | | |
| **ADOXACEAE** | | | |
| *Sambucus nigra* L. | | | X |
| *Viburnum dentatum* L. | | | X |
| **ANACARDIACEAE** | | | |
| *Toxicodendron radicans* (L.) Kuntze | | | X |
| *Toxicodendron vernix* (L.) Kuntze | | X | X |
| **APIACEAE** | | | |
| *Cicuta bulbifera* L. | X | X | X |
| *Cicuta maculata* L. | | | X |
| *Sium suave* Walt. | X | X | X |
| **AQUIFOLIACEAE** | | | |
| *Ilex verticillata* L. | X | X | X |
| **APOCYNACEAE** | | | |
| *Asclepias incarnata* L. | X | X | X |
| **ASTERACEAE** | | | |
| *Bidens connata* Muhl. ex Willd. | X | X | X |
| *Bidens frondosa* L. | X | X | X |
| *Doellingeria umbellata* (Mill.) Nees | | | X |

| | | | |
|---|---|---|---|
| *Erechtites hieraciifolius* (L.) Raf. ex DC. | | | X |
| *Eupatorium perfoliatum* L. | X | X | X |
| *Euthamia graminifolia* (L.) Nutt. | X | X | X |
| *Eutrochium dubium* (Willd. ex Poir.) E.E. Lamont | | X | |
| *Symphyotrichum lanceolatum* (Willd.) Nesom | X | | X |
| *Symphyotrichum racemosum* (Elliott) Nesom | X | X | X |
| **BALSAMINACEAE** | | | |
| *Impatiens capensis* Meerb. | X | X | X |
| **BETULACEAE** | | | |
| *Alnus incana* (L.) Moench | X | X | X |
| **BRASSICACEAE** | | | |
| *Cardamine bulbosa* (Schreb. ex Muhl.) B.S.P. | | | X |
| *Cardamine pensylvanica* Muhl. ex Willd. | | | X |
| *Rorippa palustris* (L.) Besser | | | X |
| **CABOMBACEAE** | | | |
| *Brasenia schreberi* J.F. Gmel. | | X | X |
| **CAMPANULACEAE** | | | |
| *Campanula aparinoides* Pursch | | X | X |
| **CAPRIFOLIACEAE** | | | |
| *Lonicera morrowii* Gray | | | X |
| **CONVOLVULACEAE** | | | |
| *Calystegia sepium* (L.) R. Br. | | | X |
| **CORNACEAE** | | | |
| *Swida amomum* (P. Mill.) Small | X | X | X |
| *Swida sericea* (L.) Holub | X | X | |
| **DROSERACEAE** | | | |
| *Drosera intermedia* Hayne | X | X | |
| **ERICACEAE** | | | |
| *Lyonia ligustrina* (L.) DC. | X | X | X |
| *Vaccinium corymbosum* L. | X | X | X |
| *Vaccinium macrocarpon* Aiton | X | X | |
| **HALORAGACEAE** | | | |
| *Proserpinaca palustris* L. | X | X | X |
| **HYPERICACEAE** | | | |
| *Hypericum boreale* (Britton) Bicknell | X | X | X |

| | | | |
|---|---|---|---|
| *Hypericum canadense* L. | X | | |
| *Hypericum dissimulatum* Bicknell | X | | |
| *Hypericum ellipticum* Hook. | X | X | |
| *Hypericum mutilum* L. | X | | |
| *Triadenum fraseri* (Spach) Gleason | X | X | X |
| *Triadenum virginicum* (L.) Raf. | | | X |
| **LAMIACEAE** | | | |
| *Lycopus americanus* Muhl. ex W. Barton | X | | X |
| *Lycopus uniflorus* Michx. | X | X | X |
| *Mentha arvensis* L. | | | X |
| *Scutellaria galericulata* L. | X | X | X |
| *Scutellaria lateriflor* a L. | | | X |
| **LENTIBULARIACEAE** | | | |
| *Utricularia gibba* L. | X | | |
| *Utricularia vulgaris* L. | | X | X |
| *Utricularia minor* L. | X | X | |
| **LYTHRACEAE** | | | |
| *Decodon verticillatus* (L.) Elliott | | | X |
| *Lythrum salicaria* L. | X | X | X |
| **MYRICACEAE** | | | |
| *Morella caroliniensis* (P. Mill.) Small | X | X | |
| **MYRSINACEAE** | | | |
| *Lysimachia terrestris* (L.) B.S.P. | X | X | X |
| **NYMPHAEACEAE** | | | |
| *Nuphar lutea* (L.) Sm. | X | X | X |
| *Nymphaea odorata* Aiton | | | X |
| **ONAGRACEAE** | | | |
| *Epilobium ciliatum* Raf. | | X | X |
| *Epilobium palustre* L. | X | | |
| *Ludwigia palustris* (L.) Elliott | X | X | X |
| **OROBANCHACEAE** | | | |
| *Agalinis purpurea* (L.) Pennell | X | X | X |
| *Orobanche uniflora* L. | | | X |
| **OXALIDACEAE** | | | |
| *Oxalis dillenii* Jacq. | | | X |

| | | | |
|---|---|---|---|
| **PENTHORACEAE** | | | |
| *Penthorum sedoides* L. | X | X | X |
| **PHYRMACEAE** | | | |
| *Mimulus ringens* L. | X | X | X |
| **PLANTAGINACEAE** | | | |
| *Callitriche palustris* L. | X | X | |
| *Chelone glabra* L. | | X | |
| **POLYGONACEAE** | | | |
| *Persicaria amphibia* (L.) Gray | X | X | X |
| *Persicaria arifolia (* L.) Haroldson | X | | |
| *Persicaria hydropiper* (L.) Opiz | | | X |
| *Persicaria lapathifolia* (L.) Gray | X | X | X |
| *Persicaria pensylvanica* (L.) Maza | | X | X |
| *Persicaria punctata* (Elliott) Small | X | X | X |
| *Persicaria sagittata* (L.) Gross | X | X | X |
| *Rumex crispus* L. | X | | X |
| *Rumex pallidus* Bigelow | | X | |
| *Rumex verticillatus* L. | | X | X |
| **RANUNCULACEAE** | | | |
| *Clematis virginiana* L. | | | X |
| *Ranunculus sceleratus* L. | | X | X |
| **RHAMNACEAE** | | | |
| *Frangula alnus* Mill. | | X | X |
| **ROSACEAE** | | | |
| *Potentilla argentea* L. | | | X |
| *Rosa palustris* Marshall | | X | X |
| *Rosa multiflora* Thunb. | | | X |
| *Spiraea alba* Du Roi | X | X | X |
| *Spiraea tomentosa* L. | X | X | X |
| **RUBIACEAE** | | | |
| *Galium palustre* L. | X | X | X |
| **SALICACEAE** | | | |
| *Salix eriocephala* Michx. | | X | |
| *Salix lucida* Muhl. | | X | |
| *Salix nigra* Marshall | X | X | X |

| | | | |
|---|---|---|---|
| **SAPINDACEAE** | | | |
| *Acer rubrum* L. | X | X | X |
| **SOLANACEAE** | | | |
| *Solanum dulcamara* L. | X | X | X |
| **URTICACEAE** | | | |
| *Boehmeria cylindrica* (L.) Sw. | X | X | X |
| **VERBENACEAE** | | | |
| *Verbena hastata* L. | X | X | X |
| **VIOLACEAE** | | | |
| *Viola lanceolata* L. | X | | |
| **VITACEAE** | | | |
| *Parthenocissus quinquefolia* (L.) Planch. | | | X |

## ANGIOSPERMS - MONOCOTYLEDONS

| | | | |
|---|---|---|---|
| **ALISMATACEAE** | | | |
| *Alisma subcordatum* Raf. | X | X | X |
| *Sagittaria latifolia* Willd. | X | X | X |
| **ARACEAE** | | | |
| *Lemna minor* L. | X | X | X |
| *Symplocarpus foetidus* (L.) Salisb. ex Nutt | | X | X |
| *Wolffia columbiana* Karst. | X | | X |
| **CYPERACEAE** | | | |
| *Carex alopecoidea* Tuck. | | | X |
| *Carex atherodes* Spreng. | | X | X |
| *Carex canescens* L. | | X | |
| *Carex comosa* Boott | X | X | X |
| *Carex lenticularis* Michx. | | X | |
| *Carex lupulina* Muhl. ex Willd. | X | X | X |
| *Carex lurida* Wahlenb. | X | X | X |
| *Carex pseudocyperus* L. | X | X | X |
| *Carex scoparia* Schkuhr ex Willd. | X | X | X |
| *Carex stipata* Muhl. ex Willd. | | X | |
| *Carex stricta* Lam. | X | X | X |
| *Carex utriculata* Boott | | X | X |
| *Carex versicaria* L. | | | X |

| | | | |
|---|---|---|---|
| *Carex vulpinoidea* Michx. | X | X | X |
| *Cyperus strigosus* L. | X | X | X |
| *Dulichium arundinaceum* (L.) Britton | X | | X |
| *Eleocharis acicularis* (L.) Roem. & Schult. | X | X | X |
| *Eleocharis elliptica* Kunth | | X | |
| *Eleocharis obtusa* (Willd.) Schult. | X | | X |
| *Eleocharis palustris* (L.) Roem. & Schult. | X | X | X |
| *Eleocharis tenuis* (Willd.) Schult. | X | | |
| *Rhynchospora capitellata* (Michx.) Vahl | X | | |
| *Schoenoplectus acutus* (Muhl. *ex* Bigelow) A. & D. Love | | | X |
| *Schoenoplectus pungens* (Vahl) Palla | X | X | X |
| *Schoenoplectus tabernaemontani* (K.C. Gmel.) Palla | X | X | X |
| *Scirpus atrocinctus* Fernald | X | X | |
| *Scirpus cyperinus* (L.) Kunth | X | X | X |
| *Scirpus hattorianus* Makino | X | | X |
| **HYDROCHARITACEAE** | | | |
| *Najas gracillima* (A. Braun ex Engelm.) Magnus | | X | X |
| *Najas minor* All. | X | | X |
| *Vallisneria americana* Michx. | | X | X |
| **IRIDACEAE** | | | |
| *Iris versicolor* L. | X | X | X |
| *Sisyrinchium atlanticum* Bicknell | | X | |
| **JUNCACEAE** | | | |
| *Juncus acuminatus* Michx. | | X | X |
| *Juncus articulatus* L. | X | | |
| *Juncus canadensis* Gay ex Laharpe | X | | X |
| *Juncus effusus* L. | X | X | X |
| *Juncus tenuis* Willd. | | | X |
| **POACEAE** | | | |
| *Calamagrostis canadensis* (Michx.) Beauv. | X | X | X |
| *Echinochloa crus-galli* (L.) Beauv | X | | X |
| *Glyceria borealis* (Nash) Batchelder | | X | |
| *Glyceria canadensis* (Michx.) Trin. | X | X | |
| *Leersia oryzoides* (L.) Sw. | X | | X |
| *Phalaris arundinacea* L. | X | X | X |

| | | | |
|---|---|---|---|
| *Poa palustris* L. | X | X | X |
| **PONTERIDACEAE** | | | |
| *Pontederia cordata* L. | X | X | X |
| **POTAMOGETONACEAE** | | | |
| *Potamogeton amplifolius* Tuck. | X | X | X |
| *Potamogeton bicupulatus* Fernald | | X | |
| *Potamogeton foliosus* Raf. | X | | X |
| *Potamogeton natans* L. | X | X | X |
| *Potamogeton pusillus* L. | X | X | X |
| **TYPHACEAE** | | | |
| *Sparganium americanum* Nutt. | | X | X |
| *Sparganium eurycarpum* Engelm. ex Gray | X | X | X |
| *Typha angustifolia* L. | X | X | X |
| *Typha latifolia* L. | X | X | X |
| *Typha* x *glauca* Godr. | | X | X |
| | 101 | 110 | 129 |
