# Supplementary material for: Successional dynamics of a 35 year old freshwater mitigation wetland in southeastern New Hampshire
Source: PLoS One. 2021 May 17;16(5):e0251748. doi: 10.1371/journal.pone.0251748 (PMC8128273; doi:10.1371/journal.pone.0251748)
Supplement: S2 Table — Wetland dependency ratings are based on Brooks and Croonquist 1990. (PDF) [file pone.0251748.s002.pdf]

Common Name	Scientific Name	Number of Surveys Present	Total Individuals	Proportion of Community (%)	Maximum Detection	Mean Abundance (indiv. per survey)
**Wetland Dependent & Associated Species**						
Belted Kingfisher	*Megaceryle alcyon*	1	2	0.5	2	0.2 ± 0.2
Blue-winged Teal	*Anas discors*	1	1	0.2	1	0.1 ± 0.1
Common Yellowthroat	*Geothlypis trichas*	3	4	0.9	2	0.4 ± 0.2
Double-breasted Comorant	*Phalacrocorax auritus*	1	3	0.7	3	0.3 ± 0.3
Great Blue Heron	*Ardea herodias*	4	4	0.9	1	0.4 ± 0.2
Green Heron	*Butorides virescens*	3	3	0.7	1	0.3 ± 0.2
Mallard	*Anas platyrhynchos*	3	19	4.4	9	2.1 ± 1.3
Palm Warbler	*Setophaga palmarum*	1	1	0.2	1	0.1 ± 0.1
Red-winged Blackbird	*Agelaius phoeniceus*	6	132	30.6	55	14.7 ± 7.3
Sora	*Porzana carolina*	1	1	0.2	1	0.1 ± 0.1
Spotted Sandpiper	*Actitis macularius*	3	3	0.7	1	0.3 ± 0.2
Swamp Sparrow	*Melospiza georgiana*	1	3	0.7	3	0.3 ± 0.3
Virginia Rail	*Rallus limicola*	1	2	0.5	2	0.2 ± 0.2
Wood Duck	*Aix sponsa*	3	15	3.5	7	1.7 ± 1.0
		**Individuals**	**192**	**44.8**		
		**Richness**	**14**			
**Upland Species**						
American Goldfinch	*Spinus tristis*	5	6	1.4	2	0.7 ± 0.2
American Robin	*Turdus migratorius*	1	2	0.5	2	0.2 ± 0.2
Barn Swallow	*Hirundo rustica*	1	2	0.5	2	0.2 ± 0.2
Black-capped Chickadee	*Poecile atricapillus*	5	23	5.3	7	2.6 ± 1.0
Blue Jay	*Cyanocitta cristata*	9	32	7.4	7	3.6 ± 0.6
Brown Creeper	*Certhia americana*	1	2	0.5	2	0.2 ± 0.2
Cedar Waxwing	*Bombycilla cedrorum*	1	1	0.2	1	0.1 ± 0.1
Chipping Sparow	*Spizella passerina*	2	10	2.3	8	1.1 ± 0.9
Common Grackle	*Quiscalus quiscula*	5	41	9.5	24	4.6 ± 2.6
Cooper's Hawk	*Accipiter cooperii*	1	1	0.2	1	0.1 ± 0.1
Downy Woodpecker	*Dryobates pubescens*	1	1	0.2	1	0.1 ± 0.1
Eastern Phoebe	*Sayornis phoebe*	2	2	0.5	1	0.2 ± 0.2
Gray Catbird	*Dumetella carolinensis*	9	33	7.7	8	3.7 ± 0.7
Mourning Dove	*Zenaida macroura*	4	5	1.2	2	0.6 + 0.2
Northern Cardinal	*Cardinalis cardinalis*	6	19	4.4	7	2.1 ± 0.8
Northern Mockingbird	*Mimus polyglottos*	2	2	0.5	1	0.2 ± 0.2
Pine Warbler	*Setophaga pinus*	2	2	0.5	1	0.2 ± 0.2
Red-breasted Nuthatch	*Sitta canadensis*	2	5	1.2	3	0.6 ± 0.4
Ruby-throated Hummingbird	*Archilochus colubris*	1	1	0.2	1	0.1± 0.1
Song Sparrow	*Melospiza melodia*	8	21	4.9	7	2.3 ± 0.7
Tree Swallow	*Tachycineta bicolor*	2	4	0.9	3	0.4 ±0.3
Tufted Titmouse	*Baeolophus bicolor*	3	9	2.1	5	1.0 ± 0.6
White Throated Sparrow	*Zonotrichia albicollis*	2	11	2.6	10	1.2 ± 1.1
Yellow Warbler	*Setophaga petechia*	1	1	0.2	1	0.1 ± 0.1

Yellow Rumped Warbler	*Setophaga coronata*	1	1	0.2	1	0.1 $\pm$ 0.1
		**Individuals**	**237**	**55.2**		
		**Richness**	**25**			
