# Supplementary material for: Successional dynamics of a 35 year old freshwater mitigation wetland in southeastern New Hampshire
Source: PLoS One. 2021 May 17;16(5):e0251748. doi: 10.1371/journal.pone.0251748 (PMC8128273; doi:10.1371/journal.pone.0251748)
Supplement: Attachment — Submitted filename: Response to Reviewers.pdf [file pone.0251748.s003.pdf]

30 March 2021

David G. Jenkins, PhD
Academic Editor
PLOS ONE

RE: PONE-D-21-04357
Response to Reviewers

Dear Dr. Jenkins,

On behalf of myself and my co-authors, I wish to thank you and Dr. Baldwin for your constructive comments and suggestions to improve our manuscript. In the attached pages, I detail the steps we've taken to address each recommendation, which include revised text, additional explanations, and corrections deemed necessary. These steps are organized in the manner which they were provided by yourself and Dr. Baldwin and provided line-by-line in *italics*. Line numbers in our responses and revisions to the reviewer's comments are based on the 'Revised Manuscript with Track Changes' document.

Thank you for the opportunity to provide a revised and improved manuscript which we hope will *fully* meet PLOS ONE's publication criteria.

Sincerely,

Grant McKown

cc: Gregg Moore (University of New Hampshire)
Andrew Payne (Drexel University)
Natalie White (University of New Hampshire)
Jennifer Gibson (University of New Hampshire)

<u>Reviewer 1 Comments:</u>

This is study of the long-term changes in plant communities of a 35 year old restored freshwater wetland, which is important because few studies have documented changes in freshwater vegetation for this long, particularly for woody plants. The study was carefully executed, the interpretation of the findings thoughtful, and the manuscript clearly written. Most comments I have are for clarification or to suggest improvements. No statistical tests are included, but they do not seem needed, because this is a descriptive observational study.

Specific comments of Reviewer 1:

L80-81. This sentence seems out of place and could probably be deleted – the goal of the paper is not to guide how restoration should be assessed.

Agreed. Sentence deleted (see L86 – 97).

L93. Define turnover here and in the abstract.

*Agreed. Turnover defined in the introduction (see L98 - 99). The original sentence was replaced with **"Third, the rate of species gain, loss, and turnover, measured as the rate species are lost and replaced over time, is initially high wetland creation and then declines over time."***

*In addition, clarity for the concept of turnover is included in the Abstract (see L41 – 43). The original sentence is replaced with **"Plant species composition stabilized as the rate of turnover, the measure of succession, declined by nearly half after 17 years."***

L136-37. This sentence is not clear - was there peat in the restoration site already? Maybe this was supposed to be two sentences?

*Peat was excavated from the original wetland before construction of infrastructure. The peat was then deposited and spread across the restored wetland (study site) as part of the mitigation process. The text was clarified and replaced with (see L144 – 146), "**Peat from the original wetland was excavated, containing an intact seed bank, and deposited at the restoration site. The peat was spread at a thickness of 15 – 30 cm across the restoration area."***

L138. Is the topographically restricted wetland the original wetland or the restored wetland?

*It is the restored wetland, established within the basin created by historic gravel mining at the site. Language has been added to clarify (see L148 – 149): **"Surface water runoff, direct precipitation, and groundwater are the primary water inputs into the topographically restricted restored wetland."***

L152-153. Explain how the meander survey worked. E.g., how many people, paths through the wetland, presence/absence or abundance? Also, was all vegetation sampling done the same way in 1992, 2002, and 2020?

The abundance and canopy cover of plant species were sampled with four linear transects with plots every 10 m (n = 103 plots). Two researchers conducted the plot sampling in June. The first floristic survey in 1992 (Padgett and Crow 1994) was conducted with 13 linear transects (n = 284 plots) with two researchers with no measurement of shrub or tree canopy due to the lack of shrubs and trees at the time. The locations of the transects and plots from 1992 are unknown. The second floristic survey in 2002 (Jahr and Crow 2005) did not conduct any systematic sampling (i.e., linear transects and plot sampling). They used general meander surveys and the original floristic evaluation/wetland vegetation community maps to assess qualitative (non-quantitative) changes in the species composition and area of the vegetation communities. Due to the non-systematic sampling efforts of the 2002 survey, we only compared the changes in wetland vegetation community distribution to the 1992 survey (i.e., GIS maps).

The meander survey was conducted biweekly (May – Oct) with at least two researchers following similar pathways along the perimeter of the wetland and several corridors through different vegetation communities (i.e., scrub-shrub swamp, cattails, aquatic beds). During the meander survey, only the presence/absence of each species were recorded to create the species composition list. The researchers of the 1992 floristic survey do not provide specific details on their meander surveys except for the time period of sampling (length of the growing season) and how often they conducted the surveys. The meander surveys of 2020 were comparable to both season length and how often they were conducted. The meander surveys did extend into the early fall (Sept – early Oct) compared to August of prior surveys to take into account possible late germinations in exposed, previously inundated-soils from the intense drought in the summer of 2020.

The following language was added to the results for clarity, ***"Meander survey efforts were conducted at roughly the same duration of season and frequency as 1992 and 2002 surveys."*** *(L163 – 165) and* ***"Vegetation plot sampling efforts were based on the methods of the initial 1992 survey to accurately compare the distribution of vegetation communities, however the exact locations of the transects and plots from 1992 are unknown. The visual cover and distribution of vegetation communities of 2020 were only compared to 1992, since the 2002 survey methodology did not allow for direct comparison."*** *(L174 – 176).*

L159. Is the 0.5 m2 quadrat 0.7x0.7 m square, 0.5x0.5 square (which would be 0.25 m2 in area), or a circular area of 0.5 m2 (if so what is radius as for the larger plots)?

The understory vegetation visual cover was sampled with a 0.5 m^2 square plot (0.7 m X 0.7 m). The shrub and tree canopy cover were estimated with 3 m and 5 m radius circular plots, respectively.

The sentence was revised to clarify sampling methodology (see L171 – 172). The original sentence was replaced with, ***"Visual cover was estimated to the nearest 1% (maximum of 100%) at three canopy layers: understory (0.5 m^2 square quadrat), shrubs of 2 – 5 m (3 m circular radius), and trees greater than 5 m tall (5 m circular radius)."***

L279 Table 1. Is Woody % correct or should it be number of woody species. If woody %, is it the percent of all species that were woody?

The "%" should not have been included in the row title. The row "Woody" is the percent of woody species in the entire vegetation community. The "%" was deleted from Table 1.

L281 and elsewhere. FAC species are considered to be hydrophytes by the USACE for use in identifying and delineating wetlands. *e.g.*, see Lichvar, R.W. 2013. The National Wetland Plant List: 2013 wetland ratings. Phytoneuron 2013-49: 1–241. Published 17. July 2013. ISSN 2153 733. http://www.phytoneuron.net/2013Phytoneuron/49PhytoN-2013NWPL.pdf. Please justify the inclusion of FAC species as "Upland" species.

The consideration of FAC species as "upland" species was based on (1) Certain state wetland permitting systems only consider OBL and FACW designations for mitigation performance criteria (See Spieles et al. 2006 – Community structure and quality after 10 years in two Central Ohio mitigation bank wetlands; pg. 839) and (2) Be conservative in the estimation of the number of hydrophytes in the wetland. We agree with the reviewer that the paper should follow modern classification classifications for clarity and use of results by future researchers.

The calculations for the number of hydrophytes were conducted again with "Wetland Species" containing OBL, FACW, and FAC species and "Upland Species" containing FACU and UPL species. It should be noted that the results only varied slightly with gains of "Wetland Species" by single digits in 1992 and 2002 and twelve in 2020. The time period of 2002 – 2020 still saw an increase of eight upland species, still suggesting upland species took advantage of a dough in 2017 - '18. Overall, the change in classification strengthened the case that the wetland mitigation project yielded a successful, hydrophytic vegetation community.

The updated calculations were completed for Tables 1 – 3 and their table legends. The results and discussion were updated to reflect the minor changes in the results and discussion (see L325 – 328, L494 – 499, L590).

L322-328. List the abbreviations in the table and restate their meaning here to improve ease of reading.

The abbreviations for the rate of species gain (G_p), species loss (L_p), and species turnover (T_p) are restated in the first mention of each variable to improve clarity for the audience (see L338 – 345). The abbreviations were added to Table 4 for consistency.

Table 6, 7, 8, and other place where means are reported without SD or SE (also average CoC values in Tables 1, 2, 3). Please include +/- standard deviation or standard error, and number of observation (N) for any means. These statistics will strengthen the interpretation of results, and are especially important because no statistical tests are included. Either SE or SD is reported for birds, but please also include N and clarify it the measure of variation is SE or SD.

Agreed. The +/- has been included for the Coefficient of Conservatism calculations in Tables 1 – 3 and table legends have been updated (L296; L309; L316). The +/- standard error has been included in Tables 6 and 7 for the visual cover of dominant species and table legends have been

updated (L397; L439). The use of +/- standard error is clarified in the caption of Table 8 (see L452).

L402. It is not clear how the red maple swamp supports observations of beaver activity raising water level by 1 m. I would think an increase in 1m of water would reduce tree abundance.

Agreed. The information presented about beaver activity influencing hydrology that may impact red maple swamp is not appropriate in the Results section. A revised explanation is presented in the Discussion section currently (see L521 – 523). The sentence was deleted (L419-420).

L476-478. I don't see how any of the findings presented in the paper affirm that species composition is approaching a dynamic equilibrium. This statement also seems to contradict others elsewhere about the dynamic wetland complex and shifts in the past 18 years (e.g. L 484-486).

*Agreed. The statement was intended to capture the fact the species richness is stabilizing if recent drought-related upland species are discounted from species richness gains in the past 18 years. The sentence was revised to "**If gains in these upland species are excluded, the species richness in this study seems to be stabilizing as the richness increased the same amount but in double the time (1992 – 2002 vs. 2002 – 2020).**" (see L497 – 500),*

L569-571. Explain the statement saying the wetland might not have achieved creation of scrub-shrub swamp - is this because there aren't enough shrubs?

The statement meant to convey that the area of the original scrub-shrub swamp had not been fully replaced after 35 years. It is true that isolated pockets of scrub-shrub swamp have developed in the wetland, however the area of scrub-shrub lost in 1985 has not been fully replaced after 35 years.

*The sentence was revised for clarification and reads as, "**Despite the development of approximately 0.18 ha of scrub-shrub swamp habitat within the restoration site footprint, this mitigation project has not fully replaced the original 4 ha of intact scrub-shrub swamp lost in 1985.**" (L592 – 596).*

<u>Reviewer 2 (Editor) Comments:</u>

In those review comments, I especially point to their comments regarding lines 152-3, 476-8, 569-71. I fully agree with those comments. For example, the long time interval in your study is great, but also bears the need to match methods as carefully as you can for fair comparisons through time. Please explain more fully how you did that, and to what extent methods may have changed, including how transects were located each time (e.g., randomly? matching placement in 2020?). If you matched identically your methods with earlier methods, then saying so will help greatly.

Agreed, acknowledging the differences and similarities of the methods between 1992, 2002, and 2020 will greatly clarify the effectiveness of the study. A response to this comment is provided in our response to Reviewer 1's question above.

About diagnosis of scrub-swamp status - I agree that some more explanation of that will help here. I think I see why you write that, but it just needs to be fully clear to all readers and match more closely the conclusion at the end of the Abstract.

Agreed. The last sentence of the abstract has been revised to clarify the meaning of the sentence referring to the 1:1 area of scrub-shrub swamp, not the development of the habitat itself. The sentence was revised ***"Although the mitigation project as not created the full area of lost scrub-shrub swamp after 36 years, it has developed a structurally complex habitat and diverse avian community that effectively provides the functions and values of the impacted system."*** *(see L55 – 58).*

As to my own comments (please consider them of equal weight to the reviewer's - i.e., accept or reject & rebut as you think best):

- I think the point made in Discussion at lines 553+ (about long timelines to be expected for wetland mitigation projects) could be slipped into the abstract at line 45. Right now it is factual but not connected to other outcomes cited in the Discussion. Consider that many will not read past the Abstract.

*Agreed. The sentence was deleted. Similar language was added to the Abstract, "***Researchers should consider long-term monitoring of specific sites to better understand successional patterns of created wetlands we documented long timeframes required for the development of scrub-shrub swamps, red maple swamps, and sedge meadows."*** *(see L43-L46)*

The sentence contained reference [86] (Moreno-Mateos et al. 2012 – Structural and Functional Loss in Restored Wetland Ecosystems) and was subsequently deleted. The references of [87] and [88] were renumbered appropriately in both the text and Reference section.

Other sections of the Abstract were revised to clarify the conclusions of the paper and reduce the word count. See L46+ for updated sentences and revisions.

- I find multiple spelling errors throughout the manuscript - please run a spell checker and then ensure that names are correct. For example: envrionment on line 194. Prevalance (line 197). communitiy (line 213). Paragraph following line 252: Diversitiy, Indix. These should be easy to catch with software or another reader.

The selected spelling errors have been corrected and the document has been triple-checked for spelling and grammatical errors (see minor changes scattered throughout the manuscript).

- The Shannon-Weiner index is one step from providing more useful information - please see Jost, L. (2006). Entropy and diversity. Oikos, 113(2), 363-375 for more details, but H' needs to be expressed as e raised to H'. This should also alter the calculation of Pielou's J, which is after

all a simple ratio of values. If you make this change your bird diversity data should make a bit more intuitive sense and be more current.

Thank you for the suggestion. The updated calculations were revised for both H' and J ***(Original H': 2.69, Revised H': 14.81; Original J: 0.74, Revised J: 0.38)*** *(see L449). The equation for calculating H' was updated (see L270 – 272). Reference [50] was revised to Jost 2006 to provide credit for their work (see L732-733).*

- Thanks for providing the Supporting Information tables - my bet = people will use those in the future.

Completely agree! We could not have completed this study without the supplementary tables of species presence/absence in the previous two papers on this site.

- An alternative interpretation for line 450 is that the shift toward woody species will slow down trajectories of change because they will spread more slowly than clonal graminoids etc. So then "reaching a dynamic equilibrium" may not be the case.

Agreed. The purpose of the sentence was the summarize that the species composition and species richness are stabilizing demonstrated by the declining rates in species gains, losses, and turnover. The use of "dynamic equilibrium" conveys a different ecological phenomenon. The sentence is revised as (see L497 – 500), ***"The species composition of the vegetation community is stabilizing and followed documented successional trends based on the species gain, loss, and turnover rates declining by half between 17 and 35 years post-restoration [15, 21]."***

- line 463. Does a niche exist outside of an organism? I buy the argument that it does not, and so suggest that "many small niches and" could be omitted here.

Agreed. The phrase "of many small niches and" was removed from the original sentence (see L484-485). The sentence now reads, ***"Topographical complexity within the wetland might have allowed for persistence of specialized flora."***

- line 564. Nice last paragraph - I suggest ensuring that thoughts here convey well in the Abstract.

Thank you for the suggestion. We revised the Abstract to reduce the word count and more strongly and clearly state the findings of the paper (See L41 – 58).

<u>Additional Journal Comments & Requirements:</u>

1. Please ensure that your manuscript meets PLOS ONE's style requirements, including those for file naming.

We have adhered to the style requirements of PLOS ONE.

2. In your Methods section, please provide additional location information of the study site, including geographic coordinates for the data set if available.

The location coordinates for the site were included (see L141 – 142).

3. We note that Figures 1, 2 and 3 in your submission contain [map/satellite] images which may be copyrighted. All PLOS content is published under the Creative Commons Attribution License (CC BY 4.0), which means that the manuscript, images, and Supporting Information files will be freely available online, and any third party is permitted to access, download, copy, distribute, and use these materials in any way, even commercially, with proper attribution. For these reasons, we cannot publish previously copyrighted maps or satellite images created using proprietary data, such as Google software (Google Maps, Street View, and Earth).

The imagery in Figures 1 – 3 is drone imagery captured by the research team of this study. The images are not copyrighted and can be published without any issue.
